# Pluronic F-127/Propylene Glycol Binary Building Blocks for Novel Solid Dispersion Matrix: Industrial and Ecological Paradigm to Enhance Dissolution Profile of Dapagliflozin

**DOI:** 10.3390/pharmaceutics18050560

**Published:** 2026-04-30

**Authors:** Abdelrahman Y. Sherif, Mohammad A. Altamimi, Ehab M. Elzayat

**Affiliations:** Department of Pharmaceutics, College of Pharmacy, King Saud University, Riyadh 11451, Saudi Arabia; ashreef@ksu.edu.sa (A.Y.S.); maltamimi@ksu.edu.sa (M.A.A.)

**Keywords:** novel solid dispersion matrix, thermoresponsive polymer, dapagliflozin, pluronic F-127, propylene glycol, drug amorphization

## Abstract

**Background/Objectives**: The limited aqueous solubility of therapeutically active drugs remains a significant challenge in their pharmaceutical application. This study presents a novel solid dispersion matrix (NSDM) that utilizes the inverted thermoresponsive behavior of Pluronic F127 to enhance drug dissolution while addressing the industrial and ecological limitations of conventional methods. **Methods**: For comparative assessment, a solid dispersion formulation of dapagliflozin was formulated using the NSDM approach and three conventional approaches: heat fusion (HFSD), microwave (MWSD), and lyophilization (LPSD). Differential scanning calorimetry (DSC), Fourier transform infrared spectroscopy (FTIR), and X-ray diffraction (XRD) were used to characterize the prepared formulations. In vitro dissolution test was performed to compare the pharmaceutical performance of NSDM against conventional approaches. **Results**: The NSDM exhibited a unique thermal transition to the liquid state at 32.4 °C. Moreover, the physiological assessment revealed complete liquefaction within 81.7 s. DSC and XRD confirmed amorphization of dapagliflozin in all formulations. In addition, FTIR revealed that dapagliflozin was integrated within the formulation without any chemical interaction with the excipient. Dissolution studies showed remarkable superiority of NSDM, with 97.30 ± 2.26% dissolution efficiency and a mean dissolution time of 2.40 ± 0.80 min. A multi-criteria assessment of ecological impact, worker friendliness, industrial effectiveness, and pharmaceutical performance demonstrated NSDM’s comprehensive advantages. **Conclusions:** The present approach provides a sustainable paradigm compared to conventional solid dispersion approaches. It eliminates energy-intensive operations and post-processing steps through direct capsule filling. This affords superior pharmaceutical performance while supporting sustainability and industrial applicability.

## 1. Introduction

The limited aqueous solubility of hydrophobic therapeutic molecules hinders their oral absorption and mitigates their oral bioavailability [1,2]. Among them, the solid dispersion approach has emerged as a powerful technique to improve drug solubility by amorphization of the drug within a polymeric matrix [3,4]. However, the extensive time-consuming process required by the lyophilization approach hinders its industrial application [5]. Additionally, the demand for the post-processing treatment of the obtained powder using methods such as lyophilization, microwave, fusion, etc., reduces its industrial friendliness [6,7]. Moreover, the reported retardation of drug release from the matrix of solid dispersion particles reduces the pharmaceutical benefit from a dissolution perspective [8].

Pluronic polymers have been widely used in the literature to prepare conventional solid dispersion formulations [9,10,11,12]. Their aqueous solution exhibited temperature-dependent performance. It exists as a solution under room temperatures and undergoes transitions to a semisolid state when exposed to elevated temperatures [13]. This aqueous system is typically used to facilitate the administration of aqueous formulations that convert to a matrix with sustained-release behavior in vivo [12,14]. However, inverting this transition provides a new avenue for implementing Pluronic polymers to prepare a solid dispersion with outstanding advantages. It has been reported that the thermoresponsive behavior of Pluronic polymers in a non-aqueous system has been modified compared to aqueous solvent [15]. Thus, propylene glycol will be used in the present study to induce the intended inverted thermal transition in the novel solid dispersion matrix.

The dissolution of a polymer in propylene glycol produces a liquid formulation that can be handled during industrial preparation. Furthermore, complete liquefaction at body temperature ensures total drug release from the matrix and prevents any drug entrapment within the solid dispersion. Additionally, the liquid formulation eliminates the energy-intensive post-processing operations required to obtain fine powder from solid mass using other approaches. This approach provides an industrially scalable, pharmaceutically sound, and ecologically sustainable method for preparing solid dispersion formulations that overcome the limitations of conventional techniques.

**Figure 1:**
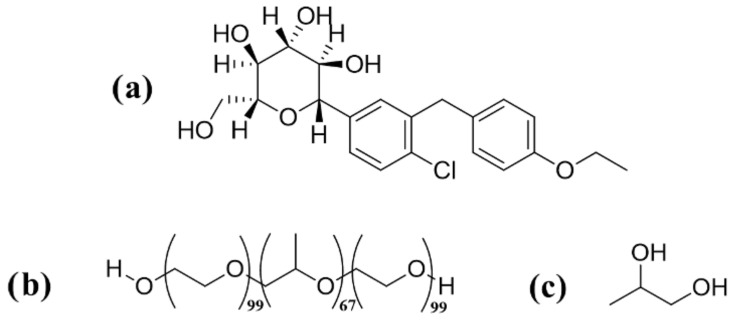
Molecular structures of (**a**) dapagliflozin, (**b**) Pluronic F127, and (**c**) propylene glycol.

This study aimed to enhance the dissolution profile of dapagliflozin (Figure 1a) using Pluronic F127 (Figure 1b) and propylene glycol (Figure 1c) using a novel solid dispersion matrix approach. For comparative assessment, three traditional approaches (heat fusion, microwave, and lyophilization) were used to prepare conventional solid dispersion formulations. The prepared novel solid dispersion matrix (NSDM) and the conventional solid dispersion (SD) were characterized using DSC, FTIR, and XRD. Moreover, an in vitro dissolution study was conducted to compare the impact of each technique on the dissolution profile of dapagliflozin.

## 2. Materials and Methods

### 2.1. Materials

Riyadh Pharma (Riyadh, Saudi Arabia) provided a therapeutic agent (dapagliflozin). Winlab Laboratory (Leicestershire, UK) provided the propylene glycol. Sigma-Aldrich (St. Louis, MO, USA) provided an acidifier agent for the preparation of the aqueous mobile phase (formic acid). AppliChem Panreac (Darmstadt, Germany) provided the organic solvent used during the preparation of the mobile phase (acetonitrile).

### 2.2. Method for Dapagliflozin Analysis

The Waters Acquity UPLC system (Milford, MA, USA) was used to quantify the concentration of dapagliflozin within the prepared samples. A reverse isocratic elution was achieved using 0.1% formic acid and acetonitrile in a ratio of 60:40, respectively. The mobile phase was eluted at a 0.25 mL/min flow rate using a connected binary solvent pump manager (Milford, MA, USA) through an Acquity UPLC BEH C18 1.7 µm (2.1 mm × 50 mm). The Acquity automatic sample manager was used to inject 2 µL, and absorption was acquired with a connected Acquity photodiode array (PDA) detector set at 222 nm.

### 2.3. Formulation Preparation

Four solid dispersion formulations were prepared using different approaches: heat fusion (HFSD), microwave (MWSD), lyophilization (LPSD), and a novel solid dispersion matrix (NSDM). The ratio of dapagliflozin to Pluronic F-127 was maintained at a fixed ratio (1:4) in all formulations to assess the effect of the preparation method on pharmaceutical performance.

#### 2.3.1. Heat Fusion Solid Dispersion (HFSD)

Dapagliflozin and Pluronic F-127 were weighed on an electronic balance at a 1:4 ratio and then placed in a porcelain mortar. The water bath was preheated to 80 ± 0.5 °C before preparing the HFSD formulation. Afterward, the porcelain mortar containing dapagliflozin and Pluronic F-127 mixture was placed for 2 min to enhance polymer melting. The molten dispersion system was mixed with a glass rod to achieve a homogeneous mixture before solidification. The obtained mixture was kept at room temperature until it solidified. Subsequently, the dispersion system was transferred to a glass mortar and crushed to get a fine powder. A uniform fine powder was collected from the sieved powder using a 315 μm mesh.

#### 2.3.2. Microwave Solid Dispersion (MWSD)

Dapagliflozin and Pluronic F-127 were weighed using an electronic balance at a 1:4 weight ratio. The MWSD formulation was obtained using a microwave instrument (Samsung Model ME0113M1). The instrument was operated at 900 W and preheated for 1 min prior to the experiment. The mixture, placed in a porcelain mortar, was irradiated for 2 min to facilitate polymer melting. The molten dispersion system was mixed with a glass rod to achieve a homogeneous mixture prior to solidification. The mixture obtained was kept at room temperature until it solidified. Subsequently, the dispersion system was crushed from the porcelain mortar wall and transferred to a glass mortar. After that, the large particles were crushed to obtain fine powder, and a uniform fine powder was collected from the sieved powder using a 315 μm mesh.

#### 2.3.3. Lyophilization Solid Dispersion (LPSD)

An aqueous solution containing dapagliflozin and Pluronic F-127 was prepared to obtain a solid dispersion via lyophilization. Briefly, Pluronic F-127 was solubilized in Milli-Q water after incubation at 4 °C to facilitate polymer solubilization. After that, dapagliflozin was added to a polymeric aqueous system and exposed to sonication to facilitate solubilization of the drug. The obtained solution was placed in a Falcon tube and frozen at −80 °C before lyophilization. The sublimation of water from frozen samples was achieved using a lyophilizer (Alpha 1–4 LD Plus, Martin Christ, Osterode am Harz, Germany). The temperature was set at −60 °C, and the lyophilization process was performed for 3 days. The resulting lyophilized formulation (LPSD) was crushed, and a uniform fine powder was collected using a sieve with a 315 μm pore size.

#### 2.3.4. Novel Solid Dispersion Matrix (NSDM)

Propylene glycol and Pluronic F-127 were used in the present study to prepare a novel solid dispersion matrix formulation containing dapagliflozin. To maintain the same drug-to-polymer ratio as in previous methods, 5% *w*/*w* dapagliflozin and 20% *w*/*w* Pluronic F-127 were incorporated into propylene glycol. The selected drug loading level (50 mg/g) is lower than the saturation solubility of dapagliflozin in pure propylene glycol (106.54 ± 1.87 mg/g). Firstly, Pluronic F-127 was added to propylene glycol, and the mixture was incubated at 50 °C until the polymer was completely dissolved. To obtain a drug-loaded formulation, dapagliflozin was added to a solution of Pluronic F-127 and propylene glycol. The mixture was sonicated for 15 min, then stirred for 10 min to facilitate the solubilization of dapagliflozin.

### 2.4. Differential Scanning Calorimetry (DSC)

Thermal analysis of dapagliflozin, Pluronic F127, and the prepared solid dispersion formulations was achieved utilizing a DSC−4000 PerkinElmer (Waltham, MA, USA) apparatus. Agents were accurately weighed in the aluminum pan and then sealed. Thermograms for analyzed samples were recorded at heating rates of 10 °C/min within the pre-determined range of (25–250 °C).

### 2.5. Fourier Transform Infrared Spectroscopy (FTIR)

Fourier transform infrared (FTIR) spectra of dapagliflozin, Pluronic F127, propylene glycol, and the prepared solid dispersion formulations were obtained using a PerkinElmer Spectrum-100 Spectrometer (PerkinElmer, Inc., Waltham, MA, USA). Data were acquired and processed utilizing Spectrum software version 6.3.5 (PerkinElmer, Inc., Waltham, MA, USA). The obtained scanning data were presented in the selected wavenumber range of 800–4000 cm^−1^.

### 2.6. X-Ray Diffraction (XRD)

The crystalline state of dapagliflozin and Pluronic F127 in the tested agents was investigated by X-ray diffraction. Dapagliflozin, Pluronic F127, and the prepared solid dispersion formulations were scanned using an X-ray diffractometer (Ultima IV, Rigaku Inc. Tokyo, Japan) equipped with Cu Kα radiation (40 kV), a scintillation counter, and a fixed monochromator. The diffraction patterns were acquired in continuous-scan mode at 1.000°/min over the angular range 3–30° (2θ). Data processing included Savitzky–Golay smoothing, Sonneveld–Visser background subtraction, and Rachinger Kα_2_ elimination. Identical instrumental parameters were maintained for all measurements to enable direct comparison of diffraction patterns. The relative degree of crystallinity of Pluronic F-127 within the drug-loaded NSDM compared to the raw polymer was estimated using the following equation:

RDC (%) = (I_sample/I_reference) × 100,

where I_sample and I_reference are the peak intensities of the drug-loaded NSDM and the Pluronic F-127, respectively.

### 2.7. Probing Native Thermal Features

The prepared novel solid dispersion matrix formulation was placed in a 10 mL glass beaker to assess its native thermal feature. After that, it was exposed to five heating and cooling cycles by placing it in an incubator set at 50 °C and a refrigerator, respectively. The conversion to the liquid and solid states was monitored visually to assess the thermal transition behavior. At the end of the fifth cycle, the glass beaker containing the formulation in solid form was inverted to determine its flowability. Images of the liquid and solid forms of the formulation were captured for documentation purposes.

### 2.8. Viscosity–Temperature Profiling

The thermal behavior of the dapagliflozin-loaded novel solid dispersion matrix was assessed by determining the liquefaction temperature. The viscosity of the drug-loaded novel solid dispersion matrix was measured as a function of temperature using a Brookfield viscometer (RVDV-II+; Brookfield Engineering Laboratories Inc., Stoughton, MA, USA) equipped with a small sample adaptor. Temperature was controlled by circulating water from a Brookfield temperature controller water bath (TC-202) through the water jacket of the small sample adaptor. The formulation was loaded into the sample chamber and equilibrated at 20 °C prior to measurement. Viscosity was measured at a fixed rotational speed of 0.5 RPM over a temperature range of 20–37 °C. The viscosity was recorded after each increasing temperature by 1.0 ± 0.1 °C.

### 2.9. Thermal Transition Time

The transition time under physiological in vivo conditions for the novel solid dispersion matrix formulation was characterized as follows. Water was preheated to 37 °C, and 100 mL was transferred to a glass beaker. The capsule filled with a novel solid dispersion matrix loaded with dapagliflozin was surrounded by a sinker to ensure complete exposure to physiological temperature. Afterward, it was placed in the glass beaker, and the time required for complete liquefaction was recorded. The physical appearance of the liquefaction process was documented through a series of captured images.

### 2.10. In Vitro Dissolution Study

The impact of used approaches on the dissolution profile of dapagliflozin was assessed using a Type II dissolution apparatus (LOGAN Inst. Corp., Somerset, NJ, USA). The pure dapagliflozin and the prepared formulation containing 10 mg of the drug were placed in a hard gelatin capsule. A wire sinker was used to enable capsule immersion during the current experiment. Phosphate buffer (pH 6.8) was used as the dissolution medium and placed in the vessel of the dissolution apparatus. The vessels filled with 900 mL were preheated to 37 ± 0.5 °C before the experiment. At zero time, the capsules were placed in the vessels, and the paddles were allowed to rotate at 50 rpm. Samples were collected using a syringe equipped with a 10-micron filter and placed directly into vials for analysis.

### 2.11. Statistical Analysis and Software

The statistical analyses were conducted using one-way ANOVA followed by Tukey’s post hoc test at *p* < 0.05 to determine the significant impact between the tested groups. Certain portions of this manuscript were drafted and/or refined with the assistance of Claude (a large language model developed by Anthropic). However, the authors held full responsibility for the overall conceptualization and findings.

## 3. Results

### 3.1. Method for Dapagliflozin Analysis

**Figure 2:**
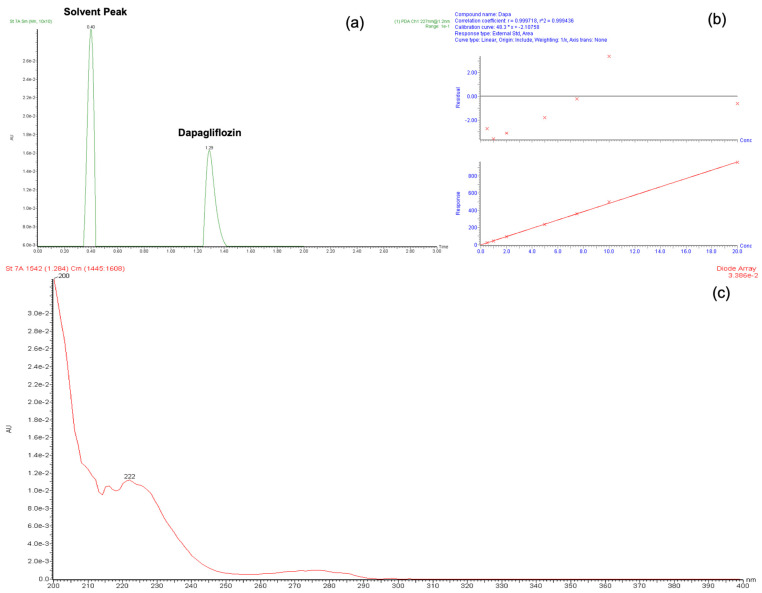
(**a**) UPLC chromatogram displaying the dapagliflozin peak resolved from the mobile solvent peak. (**b**) The calibration curve shows theoretical drug concentration plotted against measured peak areas. (**c**) UV absorption spectrum of the dapagliflozin.

The used UPLC method successfully separated the dapagliflozin peak from the solvent peak, with a retention time of 1.29 min (Figure 2a). Drug absorbance was measured at 222 nm, which corresponds to the characteristic λmax of dapagliflozin (Figure 2b). Figure 2c shows the calibration curve constructed from the injected samples, ranging from 0.5 to 20 μg/mL, with linearity indicated by a regression coefficient (r^2^) of 0.9994. The estimated linear regression equation (y = 48.3x + 2.10758) was implemented to determine the concentration of dapagliflozin within the analyzed samples.

### 3.2. Characterization of Prepared Conventional Solid Dispersion Formulations

The prepared solid dispersion formulations using conventional methods (heat fusion, microwave, and lyophilization) were characterized in terms of DSC, FTIR, and XRD.

#### 3.2.1. DSC

**Figure 3:**
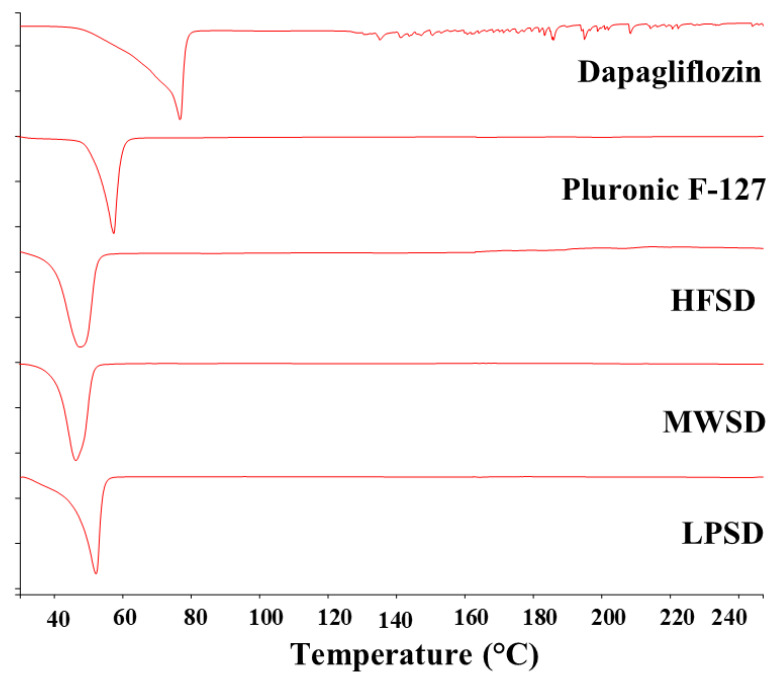
DSC thermograms of dapagliflozin, Pluronic F-127, and solid dispersions prepared by heat fusion (HFSD), microwave (MWSD), and lyophilization (LPSD) methods.

Thermal analysis for the pure dapagliflozin, Pluronic F-127, and the conventional solid dispersion formulations using three approaches is presented in Figure 3. The broad nature of dapagliflozin propanediol at 76.8 °C is consistent with the reported thermal behavior of dapagliflozin (2S)-propylene glycol monohydrate. This may be attributed to concurrent desolvation of propanediol moiety and water loss during the heating [16]. Moreover, a sharp endothermic peak for Pluronic F-127 was observed at 58.1 °C, indicating the crystalline nature of the used polymer. The results obtained agreed with previously reported studies in the literature [17,18], indicating the purity of the agents used. The results showed that the DSC thermogram of solid dispersion formulations exhibited a single endothermic event in the range of 46.47–51.29 °C, which is lower than that of pure Pluronic F-127 (58.1 °C). This could be ascribed to the integration of dapagliflozin into the solid dispersion matrix at the molecular level [19,20]. Moreover, the broad endothermic event characteristic of dapagliflozin propanediol was not detected in any of the solid dispersion formulations. Therefore, it is expected that dapagliflozin was present in amorphous form within the prepared solid dispersion formulations using all three methods. However, FTIR and XRD were performed to provide clear evidence of possible interactions between the two agents in their solid state within the formulations, respectively.

#### 3.2.2. FTIR

**Figure 4:**
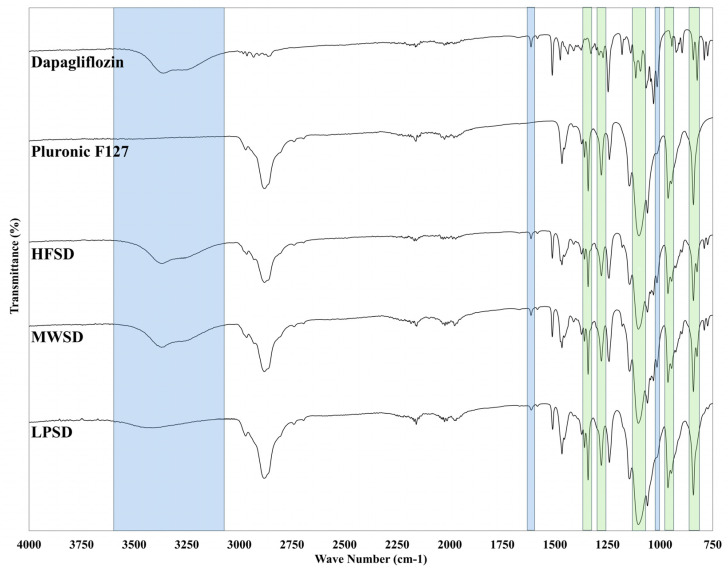
FTIR spectra of dapagliflozin, Pluronic F-127, and solid dispersions prepared by heat fusion (HFSD), microwave (MWSD), and lyophilization (LPSD) methods.

FTIR spectra were performed for dapagliflozin and the polymer used (Pluronic F127) to identify non-interfering distinctive peaks for both agents. The FTIR spectrum presented in Figure 4 reveals that dapagliflozin exhibits characteristic bands (indicated by blue color) at 3362 cm^−1^ corresponding to O-H stretching, along with distinctive peaks at 1614 and 1018 cm^−1^ corresponding to C=C stretching, and C–O bond, respectively [17]. Regarding the Pluronic F127 spectrum, the observed characteristic peaks were highlighted in green. The CH_2_ wagging doublet at 1360 and 1342 cm^−1^ and the CH_2_ twisting vibration at 1279 cm^−1^ correspond to the crystalline order and trans conformation characteristic of the pluronic F127 PEO terminal blocks, respectively [15,21]. In addition, the CH_2_ rocking vibrations at 961 and 841 cm^−1^ are additional markers associated with the ordered helical structure [15,21]. Furthermore, the C–O–C stretching triplet at 1145, 1100, and 1060 cm^−1^ is diagnostic of the 7_2_ helical backbone conformation in crystalline PEO [21,22]. The observed interfering peak corresponding to C–O stretching, at 1247 cm^−1^ for dapagliflozin and 1241 cm^−1^ for Pluronic F127, was excluded from the comparative assessment due to spectral overlap.

The current results showed that the spectrum of the dapagliflozin O-H stretching band was retained in the solid dispersion prepared by microwave and heat fusion, with a value of 3370 cm^−1^. This finding indicates that dapagliflozin disperses and shows no potential for H-bonding with Pluronic F127. On the other hand, the observed blue shift for the characteristic O-H stretching band of dapagliflozin in the FTIR spectrum of solid dispersion prepared using the lyophilization method from 3362 to 3421 cm^−1^. This could be ascribed to the molecular dispersion of the drug in the aqueous medium prior to freezing, with no opportunity for the reformation of the intermolecular hydrogen bond during sublimation. This contrasts with direct contact throughout processing between the melted drug–polymer mixture in the case of heat-fusion and microwave methods. However, the detection of the remaining characteristic peaks of dapagliflozin and Pluronic F127 in all prepared formulations suggests that there is no chemical interaction between the two agents.

#### 3.2.3. XRD

**Figure 5:**
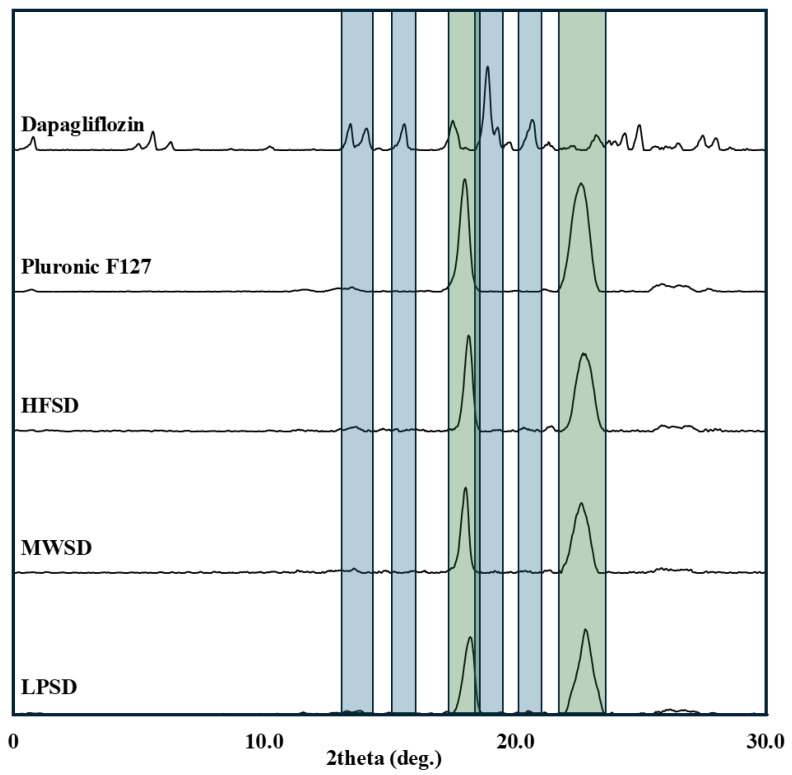
XRD spectra of dapagliflozin, Pluronic F-127, and solid dispersions prepared by heat fusion (HFSD), microwave (MWSD), and lyophilization (LPSD) methods.

Figure 5 displays the X-ray diffraction spectra of dapagliflozin, Pluronic F-127, and solid dispersions prepared by three conventional methods. The obtained data revealed that dapagliflozin, in agreement with previously published studies [16], exhibits multiple sharp diffraction peaks at 2θ values between 15° and 25° (indicated in blue color). Moreover, the XRD spectrum of Pluronic F127 exhibits two predominant sharp peaks at 19.2° and 23.3° (indicated by the green color) that agree with previously published data [23]. The characteristic diffraction peaks of dapagliflozin were completely absent in the XRD spectra of the three solid dispersion formulations (HFSD, MWSD, and LPSD) prepared by conventional methods. This provides a clear indication of the molecular dispersion of the drug in the amorphous state within the solid matrix [24,25,26]. Moreover, the presence of two characteristic diffraction peaks for Pluronic F-127 indicates its conversion to a crystalline state following cooling using heat fusion and microwave methods, and during water sublimation during lyophilization.

### 3.3. Selection of Novel Solid Dispersion Matrix Components

The Pluronic F127 aqueous solution transitioned from a liquid to a solid matrix upon exposure to body temperature [27]. This resulted in the prolonged release of entrapped agents over an extended period of time [28,29]. However, solid dispersion formulations are designed to remain in a solid state during storage and to enhance the dissolution of drugs in physiological media following the dissolution of the polymeric matrix in which the drug is present. Therefore, inversion of the thermoresponsive behavior facilitates the preparation of a novel formulation that forms a solid matrix at room temperature (25 °C) during storage, in which the drug is completely dissolved. However, once it is exposed to a slightly higher body temperature (37 °C), the matrix melts, and the polymers form a micellar structure in aqueous media. This facilitates the solubilization of the loaded drug in the GIT and its bioavailability through the concentration gradient [30].

#### 3.3.1. Selection of Non-Aqueous Solvent

Various non-aqueous solvents were used to solubilize Pluronic F127 and to prepare the novel solid dispersion matrix with the intended inverted thermoresponsive behavior. Table 1 summarizes the selected non-aqueous solvents to modulate the Pluronic F127 behavior. Pluronic F127 was mixed with non-aqueous solvents to facilitate its solubilization before testing the intentionally inverted thermoresponsive behavior. However, glycerol is a hydrophilic molecule owing to the attachment of a hydroxyl group to three carbons within the propane backbone. Therefore, Pluronic F127 failed to dissolve in glycerol due to the amphiphilic nature of Pluronic F127. It contains polyethylene oxide and polypropylene oxide, with hydrophilic and hydrophobic properties, respectively. On the other hand, Pluronic F127 dissolved in propylene glycol and propanol when incubated in an oven at 50 °C. The solubilization of Pluronic F127 in both non-aqueous solvents could be achieved through the existence of hydrophilic (OH) and hydrophobic (CH3) groups. Non-aqueous solvents containing solubilized Pluronic F127 were placed in the refrigerator to assess their tendency for solidification after exposure to lower temperature. In contrast to propanol, the system containing propylene glycol as a non-aqueous solvent solidified after cooling.

#### 3.3.2. Mechanism of Physical Transition

**Figure 6:**
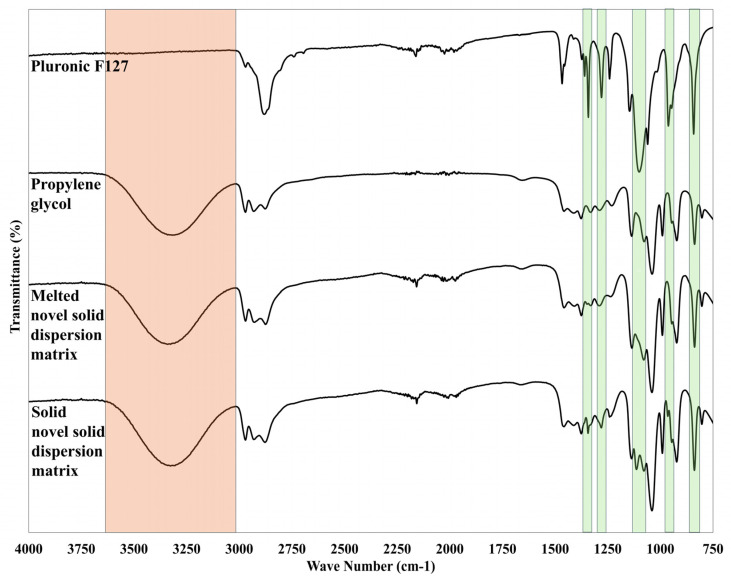
FTIR spectra of Pluronic F-127, propylene glycol, and the melted and solid novel solid dispersion matrix.

FTIR analysis was performed for Pluronic F127, propylene glycol, and the novel solid dispersion matrix after exposure to cooling and heating. Figure 6 shows the Pluronic F127 peaks (indicated by green color) and propylene glycol (indicated by orange color). The characteristic peaks for Pluronic F127 were clearly described in Section 3.2.2. However, the propylene glycol spectrum displays a distinctive band at 3319 cm^−1^, representing the reported hydrogen bond interaction between the hydroxyl groups of propylene glycol [31]. The solubilization of Pluronic F127 within propylene glycol following the melting of the novel solid dispersion matrix causes the disappearance of characteristic crystallinity markers. The expected loss in PEO crystalline order was confirmed by the absence of the CH_2_ wagging doublet at 1360 and 1342 cm^−1^ in melted NSDM formulation [15,21]. Additionally, the FTIR spectrum revealed a complete absence of the C–O–C stretching band at 1101 cm^−1^ while the remaining triplet components at 1146 and 1060 cm^−1^ were masked by propylene glycol absorptions at 1136 and 1039 cm^−1^. In addition, the CH_2_ rocking vibration at 962 cm^−1^ disappeared upon liquefaction, whereas the band at 842 cm^−1^ was retained due to overlap with the propylene glycol absorption at 838 cm^−1^. These spectral changes collectively confirm the disruption of the PEO 7_2_ helical conformation upon thermal transition [15,21]. On the other hand, the solid NSDM spectrum retained the CH_2_ wagging at 1343 cm^−1^ and the C–O–C stretching at 1114 cm^−1^. This indicates that Pluronic F127 chains maintain detectable crystalline order in the solid state.

**Figure 7:**
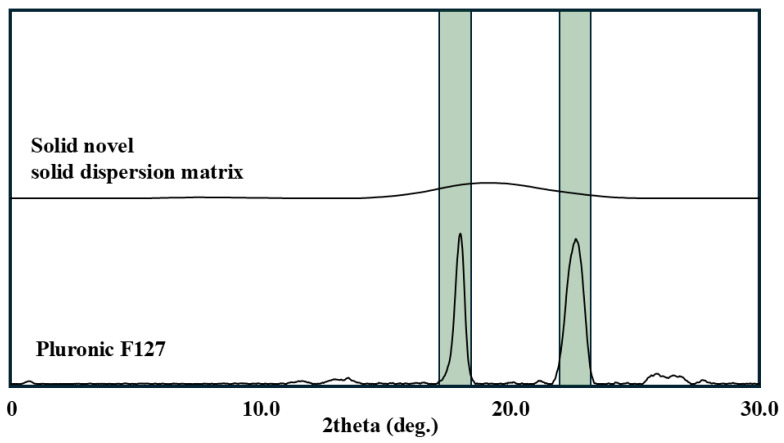
XRD spectra of Pluronic F-127 and drug-free novel solid dispersion matrix.

The Pluronic F127 within the drug-free NSDM was further investigated by XRD to provide a clear insight into its crystalline state (Figure 7). The XRD spectrum of Pluronic F127 exhibits the two predominant sharp peaks at 19.2° and 23.3° (indicated by the green color), which match with formerly published data [23]. This corresponds to the crystallographic planes of the PEO crystal lattice [22,32]. On the other hand, the absence of crystalline peaks for the PPO block is attributed to its reported inherent amorphous state [33]. However, the drug-free novel solid dispersion matrix exhibited a flat diffraction pattern with complete disappearance of both characteristic Pluronic F127 peaks. This substantial disappearance of characteristic peaks confirms that propylene glycol is the primary agent responsible for disrupting Pluronic F127 crystallinity via the hydrogen-bonding mechanism described in the FTIR analysis [15,34]. In contrast to traditional solid dispersion, the absence of sharp crystalline peaks indicates the disorder in the original crystalline nature of Pluronic F127 within NSDM [35,36]. This agrees with previously published data showing that PEO-based systems form strong hydrogen bonds with polyol solvents. This increases chain mobility and prevents the cooperative chain folding required for crystallization [37].

#### 3.3.3. Exploring Native Thermal Features

**Figure 8:**
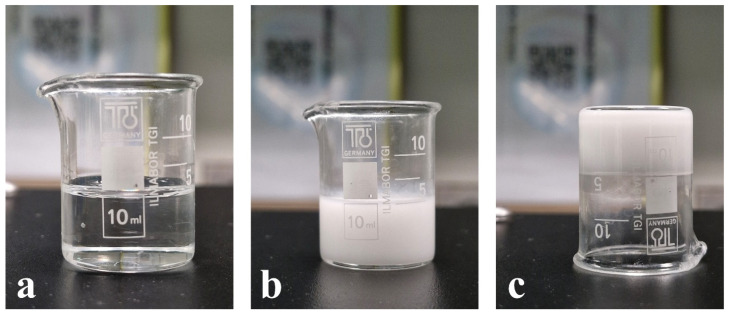
Physical appearance of novel solid dispersion matrix after exposure to (**a**) heating and (**b**) cooling. (**c**) Flipped novel solid dispersion matrix with no flow tendency.

The prepared novel solid dispersion matrix was subjected to five cycles of cooling and heating to assess its inherent tendency toward inverted thermoresponsive behavior. Figure 8a,b show the novel solid dispersion matrix after exposure to heating and cooling, respectively. Moreover, Figure 8c shows that the solid form of the Novel solid dispersion matrix withstands flipping with no tendency to flow. The present results indicate the successful formation of a novel solid dispersion matrix using thermoresponsive polymer (Pluronic F127) with inverted thermal transition behavior.

### 3.4. Characterization of Drug-Loaded Novel Solid Dispersion Matrix

The prepared novel solid dispersion matrix was loaded with dapagliflozin at the same ratio as the preparation of the three conventional solid dispersion formulations. The prepared drug-loaded Novel solid dispersion matrix was characterized by FTIR to investigate possible chemical interactions between the drug and the novel solid dispersion matrix. Moreover, XRD was used to examine the solid-state form of dapagliflozin in the novel solid dispersion matrix. After that, the thermal behavior of the Novel solid dispersion matrix was assessed in terms of melting point and physiological melting time.

#### 3.4.1. FTIR

**Figure 9:**
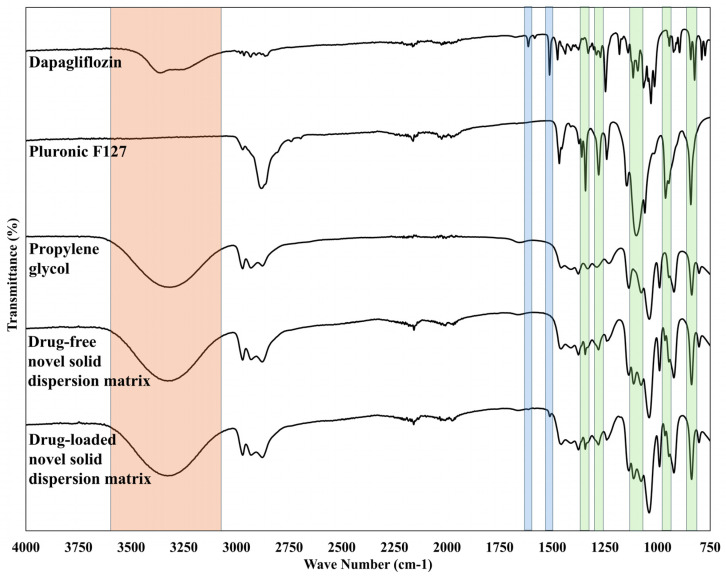
FTIR spectra of dapagliflozin, Pluronic F-127, propylene glycol, and the drug-free and drug-loaded novel solid dispersion matrix.

Figure 9 shows the characteristic peaks for dapagliflozin, Pluronic F127, and propylene glycol, which appear as blue, green, and orange, respectively. It should be noted that the hydroxyl peak for dapagliflozin was ignored due to the presence of propylene glycol in a 15-fold weight ratio compared to the drug. Therefore, the hydroxyl peak for propylene glycol was significantly more pronounced in the spectrum of the drug-loaded novel solid dispersion matrix compared to that of dapagliflozin. The presence of two peaks at 1511 and 1614 cm^−1^ within the FTIR spectrum of the drug-loaded novel solid dispersion matrix, compared to the drug-free novel solid dispersion matrix, indicates the absence of chemical interactions between the drug and other components.

#### 3.4.2. XRD

**Figure 10:**
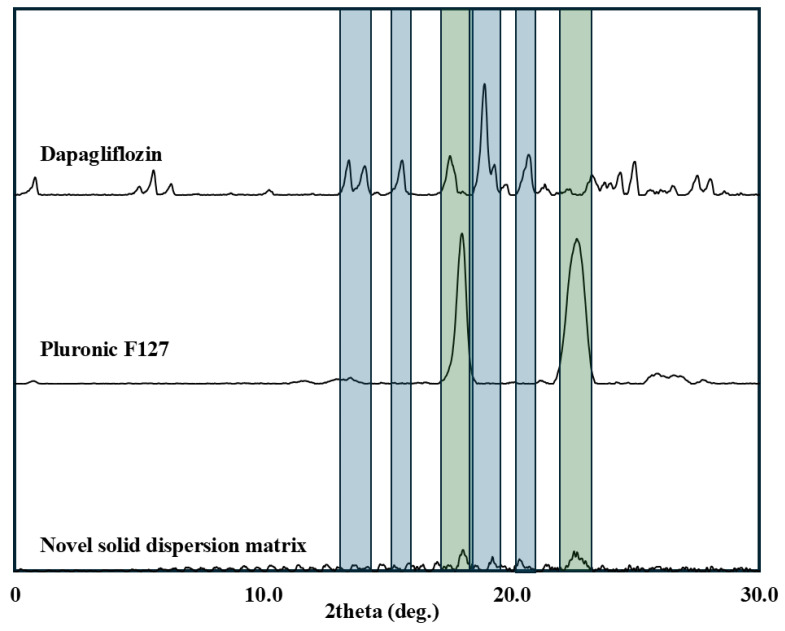
XRD spectra of dapagliflozin, Pluronic F-127, and novel solid dispersion matrix.

The crystalline state of dapagliflozin and Pluronic F127 was investigated within the prepared novel solid dispersion matrix (Figure 10). The characteristic diffraction peaks of crystalline dapagliflozin (between 15° and 25°, indicated by blue color) are completely absent. The present results confirm that the drug is present in an amorphous state within the matrix, which agrees with previously published data [38]. Moreover, the drug loading level (50 mg/g) is well below the saturation solubility in propylene glycol (106.54 ± 1.87 mg/g). This confirms that dapagliflozin is fully dissolved within the matrix in an amorphous molecular state, consistent with the XRD findings. Notably, the drug-loaded matrix retained detectable Pluronic F127 peaks at 2θ = 19.30° and 23.38°. This contrasts with the drug-free matrix, in which both peaks completely disappeared. The retention of partial PEO crystallinity in the drug-loaded system may be attributed to a competitive hydrogen-bonding mechanism in the non-aqueous propylene glycol vehicle. Dapagliflozin possesses four hydroxyl groups capable of forming hydrogen bonds with propylene glycol [16]. This drug–solvent interaction may sequester a fraction of propylene glycol hydroxyl groups into hydrogen bonds with dapagliflozin. This could reduce the availability of propylene glycol hydroxyl groups that can disrupt the PEO of poloxamer. Consequently, the crystalline 7_2_ helical conformation of PEO chains could be partially restored in the presence of dapagliflozin. For quantitative comparison, the relative crystallinity of drug-loaded NSDM was 12.1% at 2θ = 19.30° and 13.6% at 2θ = 23.38°. These findings indicate that the drug-loaded matrix retains a small residual fraction of the original PEO crystalline order, whereas Pluronic F-127 peaks are reduced in intensity rather than completely absent.

#### 3.4.3. Determination of Transition Temperature

**Figure 11:**
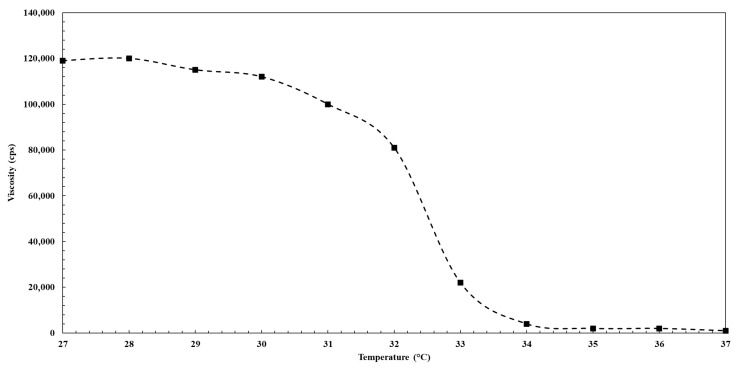
Viscosity temperature profile of drug-loaded novel solid dispersion matrix formulation.

The viscosity–temperature profile of the drug-loaded novel solid dispersion matrix (Figure 11) was investigated to quantitatively determine the temperature at which the phase transition occurs. The present results can be divided into three distinct regions based on changes in viscosity. The exhibited pronounced reduction in viscosity from 112,000 to 4000 cP in the range 30 to 34 °C could be ascribed to a dramatic transition from the solid to the liquid state. Therefore, the transition temperature was determined to be 32.4 °C, corresponding to the temperature at which the viscosity decreased by 50% between the upper and lower plateau regions. This dramatic transition could be ascribed to the progressive disruption of the intermolecular hydrogen bonds between Pluronic F-127 monomers and propylene glycol.

#### 3.4.4. Thermal Behavior Characterization

**Figure 12:**
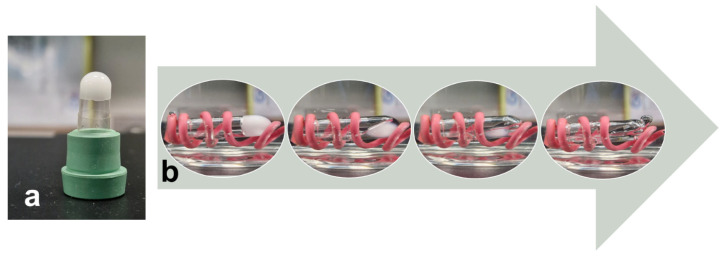
(**a**) Inverted hard gelatin capsule containing novel solid dispersion matrix. (**b**) Time-series images that show phase transition behavior of novel solid dispersion matrix upon contact with water preheated to 37 °C.

The prepared novel solid dispersion matrix formulation was assessed for thermoresponsive behavior to ensure complete liquefaction and an in vivo response to physiological temperature (37 °C). Moreover, the time to complete the transition was measured to estimate the lag time before the conversion occurs. The novel solid dispersion matrix, filled with hard gelatin capsules, was converted to a liquid state after 81.7 ± 0.6 s when placed in water heated to 37 °C. Figure 12a shows the inverted capsule filled with a novel solid dispersion matrix that withstands gravity. This indicates the prepared formulation’s ability to maintain its integrity during storage. Moreover, Figure 12b shows the transformation of the novel solid dispersion matrix during liquefaction.

### 3.5. In Vitro Dissolution Study

**Figure 13:**
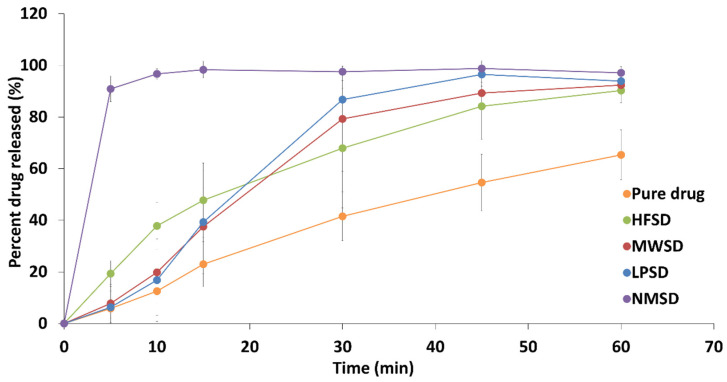
In vitro dissolution profile of pure dapagliflozin against prepared solid dispersion formulations.

The dissolution profiles of dapagliflozin release from capsules filled with solid dispersion formulations against the pure drug are presented in Figure 13. Moreover, the calculated mean dissolution time and dissolution efficiency are estimated to assess enhancements in drug release characteristics, as summarized in Table 2. The pure dapagliflozin exhibited significantly lower dissolution efficiency (39.20 ± 7.62%) with the longest mean dissolution time (25.58 ± 3.09 min). This indicates a slow dissolution rate of raw dapagliflozin. This behavior is attributed to the crystalline nature of dapagliflozin, which was previously confirmed by XRD analysis. Therefore, the amorphization approach is necessitated to enhance dapagliflozin dissolution.

The solid dispersion formulations showed significant improvements in dissolution parameters compared with pure dapagliflozin. The HFSD, MWSD, and LPSD formulations showed no statistically significant differences (*p* > 0.05) and comparable dissolution efficiencies (64.73 ± 11.08%, 64.94 ± 10.20%, and 68.89 ± 7.38%, respectively). These formulations also exhibited mean dissolution times of 18.86 ± 6.20 min, 19.62 ± 5.24 min, and 17.76 ± 5.01 min, respectively. The enhanced dissolution of dapagliflozin observed in solid dispersion formulations can be attributed to the conversion of the crystalline drug into an amorphous form, as confirmed by XRD analysis. Moreover, the presence of a hydrophilic polymer enhanced the wettability and facilitated its solubilization within dissolution media. This agrees with previously published data showing that solid dispersion can enhance drug dissolution. However, the existence of Pluronic F127 in a crystalline state within conventional three-solid dispersion formulations retards drug release. Therefore, water penetration into matrices of solid dispersion triggers gel formation that creates a viscous barrier [33].

The most interesting, statistical analysis revealed a significant (*p* < 0.05) enhancement in dissolution parameters for the novel solid dispersion matrix compared to all other formulations. It demonstrated the highest dissolution efficiency (97.30 ± 2.26%) with the shortest mean dissolution time (2.40 ± 0.80 min). This could be attributed to the inherent thermal behavior of the novel solid dispersion matrix, which completely converts to a liquid state at body temperature. This was consistent with FTIR and XRD analyses, which revealed that Pluronic F127 was present in the cross-linking matrix system with no crystalline structure. Consequently, the complete liquefaction of the novel solid dispersion matrix at body temperature prevents gel formation, eliminates the diffusion barrier, and enables the observed rapid drug release. The current findings show that our approach outperforms conventional approaches in achieving outstanding pharmaceutical performance.

### 3.6. Assessment Scores for Prepared Formulations

The prepared solid dispersion formulations were comparatively assessed across four criteria: ecological impact, worker friendliness, industrial effectiveness, and pharmaceutical performance. The following discussion provides a literature-based qualitative comparison of the four approaches.

#### 3.6.1. Ecological Impact

Energy consumption during solid dispersion preparation varies considerably across the evaluated approaches. The lyophilization process requires freezing the samples at −80 °C prior to sublimation at −60 °C under vacuum. Therefore, it is considered the most energy-intensive method among the approaches used [39,40]. On the other hand, heat fusion and microwave approaches utilize short-duration heating or radiation, respectively. This reduced the energy required to prepare the solid dispersion using these approaches. Even though a novel solid dispersion matrix requires moderate heating at 50 °C for approximately 2 h to dissolve Pluronic F127 in propylene glycol, this represents a substantially lower energy requirement than lyophilization. Regarding waste generation, the heat fusion, microwave, and lyophilization approaches produce solid powders that require additional grinding and sieving steps before capsule filling. This increases the chance of material loss during processing [41,42,43]. In contrast, the liquid nature of the novel matrix formulation minimizes waste generation owing to its ability to be directly filled into capsules without any further processing [44].

#### 3.6.2. Worker Friendliness

All four methods avoid the use of organic solvents during production, which eliminates the associated exposure risks [45]. However, the powdery nature of formulations produced by heat fusion, microwave, and lyophilization increases workers’ exposure to dust particles during grinding and sieving operations [46]. The novel matrix approach eliminates this risk entirely owing to the liquid nature of the formulation throughout the manufacturing process. Regarding explosion risk, processing under vacuum conditions during lyophilization reduces worker safety compared to other approaches [47]. In contrast, the other approach did not implement such an explosion risk, which enhances workers’ safety. For other prospective, the demand for cleaning sticky powder residues from mortar surfaces during preparation of a solid dispersion using heat fusion and microwave methods increases the risk of worker exposure [48]. Likewise, lyophilization equipment requires thorough cleaning between batches to prevent cross-contamination [39]. Conversely, the liquid nature of novel matrix formulation facilitates straightforward cleaning and reduces turnaround time between production cycles.

#### 3.6.3. Industrial Effectiveness

Capital equipment requirements differ substantially among the approaches used. The demand for lyophilizer instruments and related accessories (vacuum generators and solvent collectors) is increasing in companies’ investment. Microwave-based production at an industrial scale requires non-standard equipment that is not traditionally available in pharmaceutical manufacturing facilities. Heat fusion utilizes conventional heating equipment but necessitates additional grinding and sieving machinery. However, the availability of instrumentation used in the novel matrix approach (mixing and temperature-controlled equipment) reduces the demand for company investment. Regarding manufacturing complexity, the demand for specialized equipment during processing, in addition to grinding and sieving before filling into capsules for the lyophilization approach, reduces its industrial effectiveness and applicability. Likewise, heat fusion and microwave methods also require post-processing (grinding and sieving) before capsule filling. However, the novel matrix approach offers the lowest complexity, as the liquid formulation can be directly filled into capsules without additional processing steps. Regarding scalability, the microwave approach faces significant challenges at an industrial scale due to heterogeneous energy distribution between the surface and core of the treated powder. However, heat fusion and lyophilization require only moderate adjustments to conditions during scale-up. On the other hand, the simple mixing and heating process in the novel matrix approach avoids scale-dependent challenges. Furthermore, the novel matrix formulation’s liquid nature enables direct capsule filling without the need for lubricants. These are typically required for powder-based formulations to ensure adequate flowability during filling operations [49,50].

#### 3.6.4. Pharmaceutical Performance

Initial dissolution performance was assessed using mean dissolution time to provide a clear indication of formulation performance at the beginning of the study [51]. Moreover, the overall dissolution performance parameter was evaluated to give a clear indication of the formulation’s performance throughout the experiment [52]. The dissolution results demonstrate clear differences in pharmaceutical performance among the approaches. The novel matrix formulation exhibited the highest dissolution efficiency (97.30 ± 2.26%) with the shortest mean dissolution time (2.40 ± 0.80 min). This demonstrated significant outperformance relative to conventional approaches (*p* < 0.05). This superior performance is attributed to the complete thermal liquefaction and the elimination of the matrix barrier associated with crystalline components in other approaches [53]. However, heat-fusion, microwave, and lyophilization formulations showed comparable dissolution efficiencies (64.73–68.89%) and mean dissolution times of 17.76–19.62 min.

## 4. Conclusions

This study successfully developed and validated a novel solid dispersion matrix approach that transforms conventional solid dispersion methodology through inverted thermoresponsive behavior. The novel solid dispersion matrix system contains Pluronic F-127, in an amorphous state, which forms hydrogen bonds with propylene glycol. The rapid liquefaction of the novel formulation at physiological temperature (81.7 s) ensures complete matrix dissolution. This eliminates drug entrapment issues associated with conventional crystalline polymer matrices. This results in exceptional pharmaceutical performance, with 1.4- and 2.5-fold improvements in dissolution efficiency compared with conventional solid dispersion methods and pure dapagliflozin, respectively. Furthermore, the novel approach addresses critical industrial and ecological concerns. The novel formulation eliminates energy-intensive lyophilization processes and reduces production time from days to hours. In addition, avoiding demand for additional grinding and sieving steps minimizes dust exposure and material waste. Direct capsule-filling capability enhances scalability and manufacturing efficiency, which provides a desirable option for commercial production. This novel approach establishes a new benchmark for solid dispersion technology, harmonizing pharmaceutical performance with sustainable manufacturing practice.

## Figures and Tables

**Table 1:** Chemical structure and characteristics of the used non-aqueous solvents.

Solvent Name	Chemical Structure	Hydrophilic Part	Hydrophobic Part
Glycerol	CH_2_(OH)–CH(OH)–CH_2_(OH)	Present	Absent
Propylene glycol	CH_2_(OH)–CH(OH)–CH_3_	Present	Present
Propanol	CH_2_(OH)–CH_2_–CH_2_	Present	Present

**Table 2:** Dissolution parameters for the prepared solid dispersion formulations.

Formulation	Dissolution Efficiency (%)	Mean Dissolution Time (min)
Pure drug	39.20 ± 7.62 ^a^	25.58 ± 3.09 ^a^
HFSD	64.73 ± 11.08 ^b^	18.86 ± 6.20 ^b^
MWSD	64.94 ± 10.20 ^b^	19.62 ± 5.24 ^b^
LPSD	68.89 ± 7.38 ^b^	17.76 ± 5.01 ^b^
NSDM	97.30 ± 2.26 ^c^	2.40 ± 0.80 ^c^

Data expressed as mean ± SD (n = 3). Different letters within the same column denote significant differences (*p* < 0.05) using one-way ANOVA followed by Tukey’s post hoc test.

## Data Availability

Data are available in the manuscript.
